# Complete Genome Sequence of a New *Chickpea Chlorotic Dwarf Virus* Strain Isolated from Tomato in Kenya, Obtained from Illumina Sequencing

**DOI:** 10.1128/MRA.01344-19

**Published:** 2020-01-30

**Authors:** E. K. Avedi, C. D. Kilalo, F. M. Olubayo, I. Macharia, A. O. Adediji, E. M. Ateka, E. M. Machuka, J. M. Mutuku

**Affiliations:** a Department of Phytosanitary Services and Biosafety, Kenya Plant Health Inspectorate Service, Nairobi, Kenya; b Department of Plant Science and Crop Protection, University of Nairobi, Nairobi, Kenya; c Biosciences Eastern and Central Africa, International Livestock Research Institute, Nairobi, Kenya; d Department of Crop Protection and Environmental Biology, Faculty of Agriculture, University of Ibadan, Ibadan, Nigeria; e Department of Horticulture, Jomo Kenyatta University of Agriculture and Technology, Nairobi, Kenya; DOE Joint Genome Institute

## Abstract

High-throughput sequence analysis revealed the complete genome sequence of a novel, hitherto uncharacterized strain of *Chickpea chlorotic dwarf virus* (CpCDV) from tomato plants in Kenya. The sequence shared its highest nucleotide similarity (88.7%) with two CpCDV isolates from Burkina Faso.

## ANNOUNCEMENT

Tomato (*Solanum lycopersicum*) is one of the world’s most important vegetable crops. However, its production is constrained by viral diseases that cause yield losses (1). Many such viruses are found in the family *Geminiviridae* and the genus *Mastrevirus* with single-stranded DNA genomes (2). Seven *Mastrevirus* species infect dicotyledonous plants, including *Chickpea chlorotic dwarf virus* (CpCDV) (3–9). Nineteen CpCDV strains (strains A to S) have been described (9–11), but the presence of the virus in East Africa has not yet been reported. In this study, we identified a novel CpCDV strain, infecting tomato in Kenya, through metagenomic sequencing and phylogenetic analysis.

In 2018, five leaf samples were collected from tomato plants exhibiting stunting, yellowing, and leaf deformation in Naivasha, Nakuru County, Kenya. These samples were stored at −80°C, and total genomic DNA was extracted according to a protocol described previously (12). Genomic DNA was pooled into one sample, Tom54, for library preparation using the Nextera DNA kit. Sequencing was performed on a MiSeq system using a v.3 kit (Illumina), with paired-end reads (2 × 301 cycles). Quality control of reads was performed using FastP software (v.0.20.0), and reads were mapped to the tomato genome (GenBank RefSeq accession number GCA_000188115.3) using Bowtie (v.2.3.4.3). Unmapped reads were assembled into contigs using MEGAHIT (v.1.1.3), and those representing single-stranded DNA sequences were verified using the Kaiju virus database (13). These contigs were subjected to BLASTN 2.9.0+ searches (14), while multiple sequence alignment was performed using ClustalW in BioEdit (v.7.2.3) (15). A maximum likelihood phylogenetic tree was constructed using the Jukes-Cantor model, as implemented in MEGA (v.6.06) (16). Protein prediction of open reading frames (ORFs) was performed using ORFfinder (https://www.ncbi.nlm.nih.gov/orffinder), and virus sequence similarities were determined using SDT (v.1.2), with pairwise gap deletions (17).

**FIG 1:**
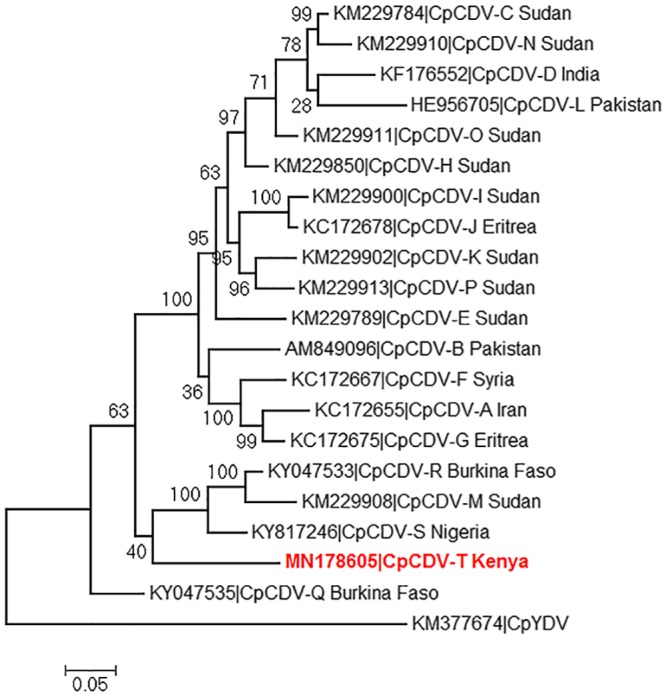
Maximum likelihood phylogenetic tree of the full genome of a novel *Chickpea chlorotic dwarf virus* strain from tomato in Kenya (CpCDV-T). Representative sequences of other CpCDV strains (CpCDV-A to CpCDV-S) were selected. The tree was rooted with an isolate of *Chickpea yellow dwarf virus* (CpYDV). The tree was drawn using MEGA (v.6.06).

Tom54 yielded 320,207 reads (Q30, 97%), which were trimmed to 314,556 reads with an average length of 174 bp. *De novo* genome assembly yielded two contigs; one was 2,469 nucleotides long, with a G+C content of 51.73%, and the other was <138 bp long. A BLASTN-based search revealed both contigs to represent CpCDV, with the larger contig sharing the highest nucleotide similarity (88.7%) with the complete circular genome sequences of two CpCDV strains infecting tomato in Burkina Faso (GenBank accession numbers KY047532 and KY047533) (11). Four ORFs, typical of *Mastrevirus* sp. genomes, were identified, i.e., V1, V2, C1, and C2. The V1 coat protein ORF encoded 283 amino acids, whereas V2 encoded the putative movement protein of 100 amino acids. C1 and C2 encoded the replication-associated proteins A and B via transcript splicing, with 302 and 143 amino acids, respectively. Phylogenetic analyses revealed a divergence of this isolate into a distinct and previously unreported clade, sharing a common ancestor with CpCDV strains M, R, and S (Fig. 1). Based on these properties, the larger contig was designated a complete, novel CpCDV genome and was deposited in GenBank under accession number MN178605.

According to the species demarcation criteria for *Mastrevirus* spp. (18), our sequence qualifies to be considered to represent a distinct CpCDV strain; therefore, we propose the name CpCDV-T. Although Kenya shares a border with Sudan, where several CpCDV isolates have been reported (8), the high level of similarity of our isolate to one from West Africa suggests a possible introduction via trade. To our knowledge, this is the first report of CpCDV in Kenya.

### Data availability.

The sequence described here was deposited in GenBank under accession number MN178605. Raw data were deposited under SRA accession number PRJNA556271 with SRA identification number SRR9737059, while the Tom54 sample was deposited under BioSample accession number SAMN12346850.
